# Correlates of Rural Naloxone Possession Among People Who Use Drugs: A Multi‐State Cross‐Sectional Analysis

**DOI:** 10.1111/jrh.70187

**Published:** 2026-07-25

**Authors:** P. Quincy Moore, L. Sarah Mixson, Rebecca Bolinski, David C. Colston, Hannah L. F. Cooper, Heidi M. Crane, Jackson Devadas, Judith Feinberg, Peter D. Friedmann, Rachel E. Gicquelais, Wiley D. Jenkins, Dalia Khoury, P. Todd Korthuis, Gordon Smith, Thomas J. Stopka, Ryan Westergaard, April Young, Mai T. Pho

**Affiliations:** ^1^ Department of Emergency Medicine The Permanente Medical Group Oakland California USA; ^2^ Department of Medicine University of Washington Seattle Washington USA; ^3^ Department of Population Science and Policy Southern Illinois School of Medicine Springfield Illinois USA; ^4^ Department of Health Behavior, Gillings School of Global Public Health University of North Carolina at Chapel Hill Chapel Hill North Carolina USA; ^5^ Rollins School of Public Health Emory University Atlanta Georgia USA; ^6^ Department of Behavioral Medicine & Psychiatry, School of Medicine West Virginia University Morgantown West Virginia USA; ^7^ Department of Population & Quantitative Health Sciences University of Massachusetts Chan Medical School Worcester Massachusetts USA; ^8^ Departments of Population Health Sciences and Medicine, School of Medicine and Public Health University of Wisconsin–Madison Madison Wisconsin USA; ^9^ Department of Public Health Sciences Clemson University Clemson South Carolina USA; ^10^ Center for Behavioral Health Data RTI International, Research Triangle Park North Carolina USA; ^11^ School of Medicine Oregon Health & Science University Portland Oregon USA; ^12^ Department of Epidemiology and Biostatistics, School of Public Health West Virginia University Morgantown West Virginia USA; ^13^ Department of Public Health and Community Medicine Tufts University School of Medicine Boston Massachusetts USA; ^14^ Department of Medicine University of Wisconsin Madison Wisconsin USA; ^15^ Department of Epidemiology & Environmental Health, College of Public Health University of Kentucky Lexington Kentucky USA; ^16^ Department of Medicine, Section of Infectious Diseases & Global Health University of Chicago Chicago Illinois USA

**Keywords:** harm reduction, naloxone, opioid, overdose, people who use drugs

## Abstract

**Purpose:**

Naloxone administration by laypersons is proven to reduce opioid overdose mortality. Despite legislative advances, people who use drugs (PWUD) in rural settings face unique barriers to naloxone possession. This study aims to examine naloxone possession and identify its correlates among a large, geographically diverse sample of people who either use opioids or inject drugs in rural areas.

**Methods:**

We performed a cross‐sectional analysis using Rural Opioid Initiative (ROI) data from rural counties of ten US states. Study sites administered a harmonized survey instrument of 3048 PWUD recruited from January 2018 to March 2020. The primary outcome was current naloxone possession. Potential correlates were identified through review of the literature. Data were collected on demographics, drug use behaviors, overdose experiences, access to care, and addiction treatment. Data were analyzed using descriptive statistics, bivariate associations, and multivariable prevalence ratios.

**Findings:**

Among 3008 participants included in the analysis, 36.4% reported possessing naloxone. Naloxone possession was associated with younger age, illegal income sources, past 30‐day opioid use, personal history of overdose, witnessing an overdose, knowing someone who died from an overdose, current injection drug use, and receiving syringes from syringe service programs (SSPs). No association was found between access to care and naloxone possession.

**Conclusions:**

Only 36% of high‐risk rural PWUD possessed naloxone. Factors such as injection drug use, overdose history, and SSP access increased the likelihood of naloxone possession. These findings highlight the need for targeted naloxone distribution strategies in rural areas, considering the unique barriers faced by rural PWUD.

## 1 Introduction

Naloxone administration by lay persons has been shown to be a safe and effective tool in reducing opioid‐related overdose mortality. The practice of overdose education and naloxone distribution (OEND) has been endorsed by the US Surgeon General and is included in the CDC Clinical Practice Guideline for Prescribing Opioids for Pain [1, 2]. Naloxone distribution to people who use drugs (PWUD) and their social networks started in the community setting, but efforts have been made to increase accessibility through other means and venues as the current opioid overdose epidemic has continued to surge [3].

Naloxone administration by lay persons has been shown to be a safe and effective tool in reducing opioid‐related overdose mortality. The practice of overdose education and naloxone distribution (OEND) has been endorsed by the US Surgeon General and is included in the CDC Clinical Practice Guideline for Prescribing Opioids for Pain [1, 2]. Naloxone distribution to PWUD and their social networks started in the community setting, but efforts have been made to increase accessibility through other means and venues as the current opioid overdose epidemic has continued to surge [3].

Following the lead of syringe services programs (SSPs) and other harm reduction organizations, clinicians in more traditional healthcare settings, such as primary care clinics, emergency departments, and substance use treatment settings have become increasingly engaged in OEND [4]. All fifty states have passed legislation protecting against civil, criminal, and professional liability for both prescribers of naloxone and lay persons administering naloxone [5]. Naloxone access in pharmacies has increased substantially over the last several years, firstly through statewide standing orders that allow people to obtain naloxone from pharmacies without a prescription [6]. Secondly, in March 2023, the US Food and Drug Administration approved a naloxone nasal spray for over‐the‐counter, nonprescription purchase [7, 8]. Naloxone has been available over‐the‐counter since September, 2023 [8]. Despite these policy advances, many individuals at high risk of experiencing or witnessing an opioid‐related overdose may not possess naloxone [9].

In regards to our specific sample during our study period, all study states maintained mechanisms for publicly supported naloxone distribution, including state appropriations, federal State Targeted Response (STR) grants, State Opioid Response (SOR) grants, Medicaid reimbursement, and state‐funded overdose education and naloxone distribution programs [10, 11, 12, 13]. While there was some variation to the specific legal frameworks, all states included in our sample had laws protecting prescribers and lay person use of naloxone at the time of our study [5]. All states except West Virgina had a statewide standing order for naloxone in place at the start of our study (January, 2018.) West Virgina first introduced this in November 2018 [5].

Barriers to care that place PWUD in rural settings at high risk for fatal overdose include further distances to travel when seeking medical care, longer response times from emergency medical services, heightened fear of calling 911 due to potential legal consequences, perceptions of law enforcement, and desire to avoid being identified by law enforcement [14, 15, 16, 17, 18]. Living in rural settings also presents PWUD with a number of multilevel barriers to substance use disorder (SUD) treatment, including challenges to confidentiality due to small, close‐knit communities where people are highly familiar with one another, and high‐threshold treatment program practices [19, 20]. In the rural United States, PWUD commonly witness overdose, which may underscore the importance of carrying naloxone in the community, however access to, possession of, and use of naloxone remains limited [21, 22]. The barriers and facilitators that drive rural PWUD preferences and ability to obtain naloxone may differ significantly from those of their urban and suburban counterparts [23, 24, 25]. Furthermore, methamphetamine use has been rising in rural areas, and perceived risk of overdose by PWUD may be declining due to changes in drug use patterns [26, 27]. Previous studies demonstrate that possession and use of naloxone among PWUD in rural settings is correlated with risk for overdose, drug of choice, injection drug use, history of overdose and witnessed overdose, and access to SSPs, although data are limited [25, 28, 29, 30, 31, 32]. Previous studies on overdose prevention in rural communities have largely focused on a single geographic region [23, 24, 25, 33].

In this study, we examine the people who possessed naloxone and identify correlates of naloxone possession among a large, geographically diverse sample of people who either use opioids and/ or inject drugs in rural areas of ten different US states. We hypothesized that in our rural setting, naloxone access is lower than documented in previous studies in urban settings, which is driven by access to harm reduction and substance use treatment, and drug use behaviors, including drug of choice and previous experience with overdose.

## 2 Materials and Methods

### 2.1 Study Overview and Setting

We used cross‐sectional, multi‐study data collected through the Rural Opioid Initiative (ROI), which has been previously described in the literature. ROI is a bi‐phasic multi‐cohort research consortium that aims to address opioid‐related overdose in the rural US ROI includes eight study sites in rural counties across ten states: Illinois, Kentucky, North Carolina, Northern New England (Massachusetts, New Hampshire, and Vermont), Ohio, Oregon, Wisconsin, and West Virginia [34]. Government definitions of rural vary by federal agency, which can affect population estimates and program offerings [35]. These differences are summarized on the Health Resources and Services Administration (HRSA) “Am I Rural” website [36]. Funding agencies used this website to confirm rurality and other indicators of vulnerability. Funders permitted small metropolitan areas, micropolitan areas, and metropolitan areas of < 250,000 persons to apply for funding under this initiative, as some rural communities with significant opioid‐related and intertwined HIV, HCV and overdose epidemics (e.g., Scott County, Indiana) [37] are classified in metropolitan statistical areas. Participants were recruited into the consortium from January 2018 to March 2020. All sites obtained local IRB approval for study activities and data sharing. Participants provided either written or verbal consent prior to research participation.

### 2.2 Recruitment and Eligibility

Across all study sites, eligible individuals self‐reported using any opioid “to get high” by any administration or injection of any drug in the past 30 days (referred to moving forward as “PWUD”). Minimum enrollment age was 15 years in two sites and 18 in the remaining sites.

Study participants were recruited using modified chain‐referral sampling, a technique closely derived from Respondent Driven Sampling (RDS) [38, 39, 40]. Investigators identified “seeds” who met study eligibility criteria and were invited to refer peer PWUD who were members of their networks. Seeds were selected to represent the sex, racial, and ethnic characteristics of the local population. Each seed and subsequent recruit were given the opportunity to recruit 3–6 eligible peers, with the process continuing until the sample size goal was met. Incentives were offered for recruitment ($10–$20 per peer) and study participation ($40–$60), with the specific payment varying based on study site [34].

### 2.3 Methods of Measurement

Study sites administered a harmonized survey instrument at the enrollment interview. ROI investigators identified 13 domains, including questions about drug use networks; socioeconomic status; ever and past 30‐day substance use and injection; severity of substance dependence; access to injection equipment; safe drug injection and use practices; substance use treatment; criminal justice involvement; access to health care; engagement in harm reduction programs; experience of stigma; and other measures.

### 2.4 Measures

The primary outcome of interest in these analyses was current possession of naloxone among PWUD in these rural areas. Current possession of naloxone was defined using the question “Do you currently have naloxone or Narcan with you or at home?” for which participants were allowed to select a binary (Yes or No) response.

To estimate associations of correlates with current naloxone possession, we evaluated several correlates within each of four exposure domains: (1) past 30‐day drug use behavior (drug of choice, current injection drug use, injection drug use frequency, syringe service program/needle exchange program as main source of syringes or needles, pharmacy as main source of syringes or needles); (2) experience with overdose (personal history of overdose, time since last overdose, number of personal overdoses, personal history of multiple overdoses, ever witnessed someone overdose, know someone who died from an overdose in the past 6 months); (3) access to care (currently possess health insurance or health care coverage, received medical care in the past six months, main place where medical care was received, and experienced barriers in accessing care in the past 6 months [i.e., “I could not pay;” “I was not sure where to go to get medical care;” “I did not have transportation;” “Clinic's hours of operation were not convenient;” “I was treated poorly at a clinic in the past;” “I did not want to be seen at a medical clinic;” “I don't trust doctors;” “I didn't have child care;” “I was too drunk or high;” “I was afraid they'd treat me with disrespect since I use drugs”]); and (4) addiction treatment (ever received any treatment for addiction, past 30 day treatment for addiction, past 30 day inpatient/outpatient treatment for addiction, past 30 day use of medication for opioid use disorder).

### 2.5 Statistical Analysis

Descriptive statistics were calculated to summarize demographic characteristics of ROI participants by their past 30‐day naloxone possession status (Table 1). We then estimated bivariate associations between past 30‐day substance use patterns (Table 2), variables within each of the four exposure domains (Table 3), and current possession of naloxone using chi‐squared tests and *t*‐tests. Individual‐study, multivariable prevalence ratios were estimated with robust confidence intervals (Table 4) to model the associations between the exposure domains of interest and naloxone possession [41]. Models were adjusted for age, gender, and race/ethnicity. Pooled effect estimates were calculated using traditional random‐effects meta‐analysis to account for the heterogeneity between study populations across sites [42]. A sensitivity analysis restricted to participants who reported past 30‐day opioid use was also performed. *p*‐values < 0.05 were considered statistically significant and analyses were conducted using Stata v17.0.

**TABLE 1:** Demographic characteristics of Rural Opioid Initiative Participants, by current naloxone possession.

		Current possession of naloxone	
	Overall	No	Yes
***N***	3008	1914 (64)	1094 (36)
**Age**, mean (SD)	36.1 (10)	36.8 (11)	35.0 (10)
**Male**	1716 (57)	1118 (58)	598 (55)
**Race/ethnicity**			
Non‐Hispanic White	2499 (83)	1610 (84)	889 (81)
Non‐Hispanic Black	93 (3)	71 (4)	22 (2)
Non‐Hispanic American Indian	205 (7)	101 (5)	104 (10)
Non‐Hispanic Other/Unknown	101 (3)	69 (4)	32 (3)
Hispanic^a^	110 (4)	63 (3)	47 (4)
**Education**			
Less than high school	677 (23)	447 (23)	230 (21)
High school or GED	1411 (47)	887 (46)	524 (48)
More than high school^b^	917 (30)	578 (30)	339 (31)
**Main source(s) of income** ^c^			
Traditional^d^	1192 (40)	810 (42)	382 (35)
Assistance^e^	1519 (51)	964 (50)	555 (51)
Illegal^f^	840 (28)	447 (23)	393 (36)
**Homelessness**, past 6 months	1599 (53)	1004 (52)	595 (54)
**Criminal legal involvement** ^g^	1968 (65)	1214 (63)	754 (69)
**Geographic region**			
Illinois	173 (6)	144 (8)	29 (3)
Kentucky	338 (11)	299 (16)	39 (4)
New England	583 (19)	291 (15)	292 (27)
North Carolina	346 (12)	176 (9)	170 (16)
Ohio	251 (8)	127 (7)	124 (11)
Oregon	173 (6)	129 (7)	44 (4)
Wisconsin	971 (32)	618 (32)	353 (32)
West Virginia	173 (6)	130 (7)	43 (4)

Abbreviation: SD, standard deviation.

^a^ Race/ethnicity are mutually exclusive categories. Hispanic includes everyone who is Hispanic. White, Black, American Indian, and Other/Unknown race include those who are White, Black, American Indian, or Other/Unknown race and not Hispanic.

^b^ “Some college,” “Associate degree, trade, or technical school,” “Bachelor's degree, other 4‐year college degree, or more.”

^c^ Does not add up to 100% because participants could select more than one option.

^d^ “Full‐time work (40 hrs/week),” and/or “Part‐time work.”

^e^ “Retirement check,” “Public assistance check—like TANF (Temporary Assistance for Needy Families), AFDC (Aid to Families with Dependent Children), etc., ” “Disability check, like SSI (Supplemental Security Income), military, or other,” and/or “Someone supports me.”

^f^ “Selling drugs,” “Selling sex,” and/or “Theft, shoplifting, or stealing.”

^g^ “Law enforcement stopped and searched you, your car, or your stuff,” “Arrested and booked for breaking the law,” “On probation, parole, supervised release, or community supervision,” and “In jail or prison.”

**TABLE 2:** Bivariate associations of Rural Opioid Initiative participants’ past 30‐day substance use, by current naloxone possession status.

		Current possession of naloxone		
	Overall	No	Yes	*p*‐value
***N***	3008	1914 (64)	1094 (36)	
Any opioids^a^	2579 (86)	1561 (82)	1018 (93)	**<0.001**
Number of days used, mean (SD)	15.7 (12)	13.5 (12)	19.6 (11)	**<0.001**
Methamphetamine	2241 (75)	1442 (75)	799 (73)	0.13
Number of days used, mean (SD)	12.6 (12)	12.5 (11)	12.8 (12)	0.5
Cocaine/crack	1311 (44)	761 (40)	550 (50)	**<0.001**
Number of days used, mean (SD)	4.2 (8)	3.7 (7)	5.2 (9)	**<0.001**
Benzodiazepines	1423 (47)	852 (45)	571 (52)	**<0.001**
Number of days used, mean (SD)	3.9 (7)	3.7 (7)	4.4 (7)	**0.006**
Multiple classes of drugs used^b^	2532 (84)	1550 (81)	982 (90)	**<0.001**
Number of classes, mean (SD)	2.9 (1)	2.8 (1)	3.2 (1)	**<0.001**
Simultaneous injection of an opioid and a stimulant (e.g., speedball)^c^	1018 (40)	508 (32)	510 (52)	**<0.001**
Number of days used, mean (SD)	5.0 (8)	3.7 (7)	6.9 (10)	**<0.001**

Abbreviation: SD, standard deviation.

^a^ Heroin, fentanyl, opioid pain medication, synthetic opiates, buprenorphine, and methadone used “to get high.”

^b^ Use of ≥2 drug classes (opioids [heroin, fentanyl, opioid pain medication, synthetic opiates, buprenorphine, methadone] methamphetamine, cocaine/crack, prescription anxiety drugs [not as prescribed], gabapentin, clonidine, and/or other) by any route in past 30 days.

^c^ Among participants reporting injection drug use in the past 30 days (*n* = 2554).

The bolded values represent statistically significant *p*‐values < 0.05.

**TABLE 3:** Bivariate associations of Rural Opioid Initiative participants, by experience with overdose, access to care, substance use and injection drug use behaviors, addiction treatment and current naloxone possession status.

		Current possession of naloxone		
	Overall	No	Yes	*p*‐value
***N***	3008	1914 (64)	1094 (36)	
**Experience with overdose**				
Personal history of overdose	1475 (49)	816 (43)	659 (60)	**<0.001**
Time since last overdose, years, mean (SD)	2.9 (5)	3.6 (6)	2.0 (4)	**<0.001**
Number of personal overdoses, lifetime, mean (SD)	2.8 (14)	2.6 (16)	3.0 (9)	0.4
Personal history of multiple overdoses	1077 (36)	570 (30)	507 (46)	**<0.001**
Ever witnessed someone overdose	2309 (77)	1355 (71)	954 (87)	**<0.001**
Knows someone who died from overdose, past 6 months	2165 (72)	1302 (68)	863 (79)	**<0.001**
**Access to care**				
Health insurance or health care coverage	2229 (81)	1429 (75)	800 (73)	0.2
Received Medical Care, past 6 months	2434 (81)	1527 (80)	907 (83)	0.07
Main place received medical care, past 6 months				0.1
Private doctor	821 (27)	512 (27)	309 (28)	
Community health center	471 (16)	304 (16)	167 (15)	
Health department	109 (4)	67 (4)	42 (4)	
Urgent care	295 (10)	178 (9)	117 (11)	
Emergency room	561 (19)	368 (19)	193 (18)	
Other	177 (6)	98 (5)	79 (7)	
Any barriers in accessing care, past 6 months^a^	2073 (69)	1282 (67)	791 (72)	**0.007**
Number of barriers in accessing care (out of 10), mean (SD)^a^	2.3 (2)	2.2 (2)	2.4 (2)	**0.004**
**Substance use and injection drug use behaviors**				
Drug of choice				**<0.001**
Opioids^b^	1638 (54)	896 (47)	742 (68)	
Stimulant^c^	1243 (41)	917 (48)	326 (30)	
Prescription anxiety medication	38 (1)	26 (1)	12 (1)	
Other^d^	89 (3)	75 (4)	14 (1)	
Current injection drug use, past 30 days	2554 (85)	1572 (82)	982 (90)	**<0.001**
Injection drug use frequency, past 30 days^e^				**<0.001**
Daily or more	1710 (67)	951 (61)	759 (77)	
More than weekly, less than daily	308 (12)	223 (14)	85 (9)	
Weekly	167 (7)	130 (8)	37 (4)	
Monthly	345 (14)	249 (16)	96 (10)	
SSP/NEP as main source of syringes or needles, past 30 days^f^	937 (37)	228 (28)	163 (50)	**<0.001**
Pharmacy as main source of syringes or needles, past 30 days^f^	451 (18)	443 (18)	494 (17)	0.2
**Addiction treatment**				
Ever received any treatment for addiction	2328 (77)	1442 (75)	886 (81)	**0.004**
Received any treatment for addiction, past 30 days	999 (33)	577 (30)	422 (39)	**<0.001**
Attended inpatient/outpatient treatment, past 30 days	736 (24)	414 (22)	322 (29)	**<0.001**
Received MOUD, past 30 days	564 (19)	274 (14)	290 (27)	**<0.001**

Abbreviations: NEP, needle exchange program; SD, standard deviation; SSP, syringe service program.

^a^ 10 barriers to accessing medical care assessed (“I could not pay;” “I was not sure where to go to get medical care;” “I did not have transportation;” “Clinic's hours of operation were not convenient;” “I was treated poorly at a clinic in the past;” “I did not want to be seen at a medical clinic;” “I don't trust doctors;” “I didn't have child care;” “I was too drunk or high;” “I was afraid they'd treat me with disrespect since I use drugs”).

^b^ Includes heroin, fentanyl/carfentanil, opiate painkillers, synthetic opioids, buprenorphine, and/or methadone. heroin, fentanyl/carfentanil, opiate painkillers, and/or synthetic opioids (e.g., U47700, U4, or “Pink”).

^c^ Includes cocaine/crack, and/or methamphetamine/crystal meth.

^d^ Includes gabapentin, clonidine, and/or other.

^e^ Among participants reporting injection drug use in the past 30 days.

^f^ Among participants reporting ever injecting drugs.

**TABLE 4:** Multivariate associations of Rural Opioid Initiative participants, by experience with overdose, access to care, substance use and injection drug use behaviors, addiction treatment and current naloxone possession status, adjusting for age, sex, and race/ethnicity (*N* = 3008).

		Meta‐analysis			
	*n*	PR^a^	95% CI	*p*‐value	*I* ^2^
**Experiences with overdose**					
Personal history of overdose	2930	1.47	1.34–1.62	**<0.001**	70%
Personal history of multiple overdoses	2889	1.44	1.31–1.57	**<0.001**	71%
Ever witnessed someone overdose	2977	1.97	1.68–2.31	**<0.001**	46%
Knows someone who died from an overdose in past 6 months	2900	1.29	1.13–1.47	**<0.001**	16%
**Access to care**					
Health insurance or health care coverage	2921	0.99	0.88–1.1	0.8	32%
Barriers in accessing care, past 6 months^b^	2971	1.02	0.92–1.13	0.7	0%
**Substance use and injection drug use behaviors**					
Current injection drug use, past 30 days	3006	1.53	1.30–1.81	**<0.001**	0%
Syringe service program/needle exchange program as main source of syringes or needles, past 30 days^c^	2524	1.72	1.56–1.89	**<0.001**	32%
Pharmacy as main source of syringes or needles, past 30 days^c^	2524	0.69	0.60–0.79	**<0.001**	99%
**Addiction treatment**					
Ever received any treatment for addiction	2971	1.15	1.02–1.30	**0.02**	27%
Received any treatment for addiction, past 30 days	2960	1.23	1.12–1.36	**<0.001**	37%
Attended inpatient/outpatient treatment, past 30 days	2945	1.27	1.15–1.40	**<0.001**	44%
Received MOUD, past 30 days	2932	1.46	1.32–1.61	**<0.001**	70%

Abbreviations: CI, confidence interval; PR, prevalence ratio.

^a^ Relative risk regression to estimate the prevalence ratio for exposures of interest and naloxone possession, adjusted for age, sex, race/ethnicity.

^b^ 10 barriers to accessing medical care assessed (“I could not pay;” “I was not sure where to go to get medical care;” “I did not have transportation;” “Clinic's hours of operation were not convenient;” “I was treated poorly at a clinic in the past;” “I did not want to be seen at a medical clinic;” “I don't trust doctors;” “I didn't have child care;” “I was too drunk or high;” “I was afraid they'd treat me with disrespect since I use drugs”).

^c^ Among participants reporting ever injecting drugs.

## 3 Results

Among the 3048 participants in the ROI consortium, 3008 (99%) were included in analyses; 40 participants were excluded because of missing responses to current naloxone possession. The mean age of the analytic sample was 36.1 years (SD: 10.3), 57% were male, and a majority self‐reported race as non‐Hispanic White (83%) (Table 1). Overall, 1094 (36%) participants reported currently possessing naloxone. Participants who self‐reported carrying naloxone, compared to participants who reported not carrying naloxone, were slightly younger (35.0 vs. 36.8 years, respectively, *p* < 0.001) and more likely to have an illegal source of income such as drug selling, theft, shoplifting or stealing (36% vs. 23%, *p* < 0.001).

Overall, 2579 (86%) participants reported past 30‐day opioid use (fentanyl, heroin, opiate pain medication, and/or other synthetics [e.g., U47700, U4, or “Pink”]) and 2241 (75%) participants reported methamphetamine use (Table 2). In bivariate comparisons of substance use behaviors, respondents who reported current possession of naloxone were more likely to have past 30‐day opioid use (93% vs. 82%, *p* < 0.001), and opioid use was more frequent in the past 30 days among those who currently possessed naloxone compared to those who did not (mean number of days = 19.6, standard deviation [SD] = 11.2 vs. mean number of days = 13.5 [SD] = 12.0, *p* < 0.001) (Table 2). Comparatively, there was no difference in past 30‐day methamphetamine use between participants who possessed naloxone and those who did not (Table 2).

In models adjusted for age, gender, and race/ethnicity, we found that personal history of overdose (prevalence ratio [PR] = 1.47, 95% Confidence Interval [95% CI] = 1.34–1.62), personal history of multiple overdoses (PR = 1.44, 95% CI = 1.31–1.57), ever witnessed someone overdose (PR = 1.97, 95% CI = 1.68–2.31), and knowing someone who died from an overdose in the past 6 months (PR = 1.29, 95% CI = 1.13–1.47) (Table 4) were associated with a higher prevalence ratio of currently possessing naloxone. Among drug use behavior outcomes, current injection drug use (PR = 1.53, 95% CI = 1.30–1.81) and utilizing a syringe service program or needle exchange program as the main source of syringes or needles in the past 30 days (PR = 1.72, 95% CI = 1.56–1.89) were associated with a higher prevalence ratio of currently possessing naloxone. Utilizing a pharmacy as the main source of syringes or needles in the past 30 days (PR = 0.69, 95% CI = 0.60–0.79) was associated with a lower prevalence ratio of currently possessing naloxone. However, this association was sensitive to one site‐specific estimate. In Kentucky, only 8 participants reported pharmacy as their main source of syringes, and none of these participants reported current naloxone possession, producing an extreme site‐specific estimate. In post hoc sensitivity analysis excluding Kentucky, the pooled association was attenuated and no longer statistically significant (PR = 0.90, 95% CI = 0.79–1.03 [Supplemental Figure s]), and heterogeneity was reduced from 99.0% to 45.6%. Among addiction treatment outcomes, having ever received any treatment for addiction (PR = 1.15, 95% CI = 1.02–1.30), having received any treatment for addiction in the past 30 days (PR = 1.23, 95% CI = 1.12–1.36), having attended inpatient and/or outpatient treatment in the past 30 days (PR = 1.27, 95% CI = 1.15–1.40), and having received medication for opioid use disorder (MOUD) in the past 30 days (PR = 1.46, 95% CI = 1.32–1.61) were associated with higher prevalence ratios of currently possessing naloxone. We found no association between the access to care domain and possession of naloxone.

In our sensitivity analyses, restricting participants who reported using an opioid in the past 30 days slightly attenuated the associations between exposures of interest and naloxone possession, except for knowing someone who died from an overdose in the past 6 months and current injection drug use, where associations were slightly higher. Nevertheless, all results remained consistent with our main analysis (Table S1).

## 4 Discussion

In this cross‐sectional study of naloxone possession among more than 3000 people who either misuse opioids or inject illicit drugs in rural areas across ten states, just over one third (36.4%) reported possessing naloxone, though more than 85% reported past month use of opioids and 77% had previously witnessed an overdose. This large and geographically diverse study highlights a large proportion of PWUD in rural settings that are at high risk of experiencing and witnessing opioid overdose and do not possess naloxone. Even among people who have ever witnessed someone overdose, the group with the highest prevalence ratio in our adjusted models, more than half of individuals did not have naloxone. This shortfall has significant implications as policymakers and public health officials make decisions about how best to deliver evidence‐based harm reduction interventions to PWUD in rural settings.

The low prevalence of naloxone possession in this rural population would fall on the low end of the eight studies included in a 2021 meta‐analysis not focused on rural populations that observed a 57% pooled prevalence of take‐home naloxone ownership in PWUD [9]. It is also on the low end of other studies analyzing naloxone possession in rural populations [24, 25, 28, 29, 32]. Studies focused on urban populations tended to have significantly higher naloxone prevalence than our rural sample, potentially due to better geographic access and a denser concentration of accessible and non‐alienating harm reduction services [43, 44, 45, 46, 47]. This disparity in naloxone possession by high‐risk, rural PWUD highlights the need for improved infrastructure for naloxone distribution in rural areas.

Participants who had received syringes from a SSP had a higher prevalence ratio for possessing naloxone than people who had not. These data indicate the important role played by SSPs in providing naloxone and suggest that this role should be explicitly enhanced and supported in its own right. Further, additional means and venues of distribution should be explored and evaluated given that only 36% of our sample indicated the SSP was their primary place for obtaining syringes in the past 30 days. Novel programs or venues distributing naloxone should consider how to incorporate the non‐stigmatizing, respectful, and discrete environment provided by SSPs, which, combined with the availability of naloxone and other supplies free‐of‐charge, likely make SSPs an appealing and low‐barrier source of education, support, and supplies. Despite almost all of the included states having statewide standing orders allowing pharmacies to provide naloxone without a prescription at the time our data were collected, pharmacy‐based syringe access did not appear to function as a consistent pathway to naloxone possession in our rural sample. In the primary pooled analysis, participants who reported pharmacies as their main source of syringes had a lower prevalence of naloxone possession. However, this finding was sensitive to the Kentucky site‐specific estimate, where sparse data produced an extreme estimate. After excluding Kentucky, the findings were no longer significant. Therefore, these findings should be interpreted cautiously. However,  these results may indicate that pharmacies were not consistently serving as integrated points of access for both sterile syringes and naloxone across rural settings. This is likely due to the high cost of naloxone in most pharmacies, as well as limited stock in pharmacies in some rural communities [48, 49]. Thus, careful evaluation will be necessary to examine whether those at highest risk of overdose can afford and benefit from pharmacy‐based take‐home‐naloxone as nasal naloxone becomes more widely available over‐the‐counter in pharmacies.

Outside of SSPs, we found that receipt of medical care, the specific location of care, and perceived barriers to care had no association with possessing naloxone. It is unclear whether these findings indicate that naloxone is insufficiently distributed to those who need it in traditional care settings in the study regions and/or whether patients at high risk of overdosing are inadequately identified by healthcare providers [50].

In contrast to PWUD who had accessed non‐addiction medical care, participants who had received addiction treatment, particularly during the last thirty days, were more likely to have naloxone. Opioid treatment programs (OTP) have been shown to be successful venues for OEND in both urban and rural areas [51]. Our findings showed that being treated for OUD increased the likelihood that a patient will possess naloxone, but it remains concerning that nearly half of patients being actively treated with MOUD did not have naloxone. Some of this shortfall may be explained by barriers to implementation of OEND, such as patient receptivity, cost of naloxone, staff time, and prohibitive pharmacy board regulations [52]. It is also worth highlighting that the shortage of OTPs seen nationally is magnified greatly in rural areas, which further limits the potential impact of OTPs in preventing death from overdose [53]. Improved access to OTPs in rural areas may also improve access to naloxone for high risk PWUD and their social networks.

We found that people who use opioids as their drug of choice and those who have used opioids in the last thirty days were more likely to possess naloxone. Of note, there was no association between last 30‐day methamphetamine use and naloxone possession. These individuals, who may have little to no opioid tolerance, represent an opportunity for intervention given the geographic spread of methamphetamine use and increasing presence of fentanyl found in methamphetamine samples [26, 27, 54, 55, 56]. People who use methamphetamine and opioids concomitantly have been shown to be at a significantly higher risk of overdose than those who use opioids or methamphetamine alone [54, 57, 58, 59]. Improving efforts to distribute naloxone and fentanyl test strips to rural people who use methamphetamine is a critical step toward reducing death from overdose in rural areas.

Respondents who reported an illegal source of income were more likely to possess naloxone than those who reported legal income. Specifically, PWUD with income from both drug selling as well as theft, shoplifting or stealing were more likely to have naloxone than their counterparts. The approximately 28% of participants who reported receiving income from selling drugs may represent an important group that could facilitate naloxone distribution. People who supply drugs are likely to have frequent contact with others who are at high risk for overdose, making them more likely to witness an overdose. They also represent a potential source of sterile syringes, naloxone, and other harm reduction education and materials. Evidence of this approach is scant but supports the idea that encouraging these individuals to carry or even distribute naloxone may help broaden the reach of harm reduction organizations [60].

### 4.1 Strengths and Limitations

These findings have limitations. First, the cross‐sectional nature of the study prevents inferences about causality. Second, there was heterogeneity in the enrollment approach between study sites. While inclusion criteria were largely the same, there was some minor variation between sites. Seven sites included both people who inject drugs (PWID) and/or people who used opioids, while one site included only PWID [34]. The specific venues through which identification of the initial seeds in the modified chain‐referral sampling approach also varied [34]. To control for this heterogeneity, we included geographic region in our meta‐analysis and our findings were largely consistent with those of the bivariate analyses. Some *I* ^2^ values in our multivariable regression models exceed 50%, which may reduce the robustness of the pooled results for these models. Additionally, we did not measure how often respondents that reported naloxone possession carried naloxone on their person. We acknowledge that some participants who affirmed possession may have had naloxone at home, but not readily accessible while using or observing others using drugs. Lastly, data were self‐reported and subject to recall and social desirability bias. Nevertheless, the robust size and geographic diversity of this cohort help offset some of these limitations. The previously understudied nature of opioid use and naloxone possession in the rural population also makes this article an important contribution to the literature.

## 5 Conclusions

In this ten‐state study of PWUD in rural settings, we found that only 36% of this high‐risk sample possessed naloxone. Injection drug use, history of personal or witnessed overdose, knowing someone who died from overdose in the past six months, receiving addiction treatment, and receiving syringes from an SSP were associated with an increased likelihood of possessing naloxone. Notably, use of methamphetamine was not significantly associated with naloxone possession. As policies aimed at increasing widespread naloxone distribution evolve, it is important to consider the unique factors that influence the likelihood of PWUD in rural settings to obtain and carry naloxone.

## Funding

This publication is based upon data collected and/or methods developed as part of the Rural Opioid Initiative (ROI), a multi‐study cohort with a common instrument which was developed collaboratively by investigators at eight research institutions and at the National Institute of Drug Abuse (NIDA), the Appalachian Regional Commission (ARC), the Centers for Disease Control and Prevention (CDC), and the Substance Abuse and Mental Health Services Administration (SAMHSA). Research presented in this publication is the result of secondary data harmonization and analysis and supported by grant U24DA048538 from NIDA. Primary data collection was supported by grants UG3DA044829/UH3DA044829, UG3DA044798/UH3DA044798, UG3DA044830/UH3DA044830, UG3DA044823/UH3DA044823, UG3DA044822/UH3DA044822, UG3DA044831/UH3DA044831, UG3DA044825, UG3DA044826/UH3DA044826, and U24DA044801 co‐funded by NIDA, ARC, CDC, and SAMHSA.

## Conflicts of Interest

The authors declared no competing interests.

## Supporting information




**Table S1**: Multivariate associations of Rural Opioid Initiative participants who used any opioids in the past 30 days, by experience with overdose, access to care, substance use and injection drug use behaviors, addiction treatment and current naloxone possession status, adjusting for age, sex, and race/ethnicity (*n* = 2579)
Supplemental Figures. Forest plots of regression models with *I* ^2^ scores of greater than 50%.
