# Supplementary material for: Correlates of Rural Naloxone Possession Among People Who Use Drugs: A Multi‐State Cross‐Sectional Analysis
Source: J Rural Health. 2026 Jul 25;42(3):e70187. doi: 10.1111/jrh.70187 (PMC13401094; doi:10.1111/jrh.70187)

**Supplemental Table 1.** Multivariate associations of Rural Opioid Initiative participants who used any opioids in the past 30 days, by experience with overdose, access to care, substance use and injection drug use behaviors, addiction treatment and current naloxone possession status, adjusting for age, sex, and race/ethnicity (n = 2,579)

|  |  | **Meta-analysis** | | | |
| --- | --- | --- | --- | --- | --- |
|  | **n** | **PR**^1^ | **95% CI** | **p-value** | **I^2^** |
| **Experiences with overdose** |  |  |  |  |  |
| Personal history of overdose | 2,515 | 1.43 | 1.30-1.58 | **<0.001** | 60% |
| Personal history of multiple overdoses | 2,480 | 1.39 | 1.27-1.52 | **<0.001** | 52% |
| Ever witnessed someone overdose | 2,556 | 1.85 | 1.56-2.18 | **<0.001** | 14% |
| Knows someone who died from overdose in past 6 months | 2,502 | 1.22 | 1.07-1.39 | **<0.001** | 26% |
| **Access to care** |  |  |  |  |  |
| Health insurance or health care coverage | 2,510 | 1.00 | 0.89-1.12 | 0.9 | 0% |
| Barriers in accessing care, past 6 months^2^ | 2,550 | 0.96 | 0.87-1.07 | 0.5 | 0% |
| **Substance use and injection drug use behaviors** |  |  |  |  |  |
| Current injection drug use, past 30 days | 2,577 | 1.61 | 1.35-1.91 | **<0.001** | 0% |
| SSP/NEP as main source of syringes or needles, past 30 days^3^ | 2,143 | 1.67 | 1.52-1.85 | **<0.001** | 18% |
| Pharmacy as main source of syringes or needles, past 30 days^3^ | 2143 | 0.75 | 0.66-0.86 | **<0.001** | 99% |
| **Addiction Treatment** |  |  |  |  |  |
| Ever received any treatment for addiction | 2,559 | 1.14 | 1.01-1.29 | **0.04** | 44% |
| Received any treatment for addiction, past 30 days | 2,550 | 1.19 | 1.08-1.31 | **<0.001** | 14% |
| Attended inpatient/outpatient treatment, past 30 days | 2,538 | 1.24 | 1.12-1.37 | **<0.001** | 42% |
| Received MOUD, past 30 days | 2,528 | 1.36 | 1.23-1.50 | **<0.001** | 41% |

Abbreviations: CI, confidence interval; PR, Prevalence Ratio.

^1^ Relative risk regression to estimate the prevalence ratio of the exposures of interest and naloxone possession, adjusted for age, sex, race/ethnicity.

^2^ 10 barriers to accessing medical care assessed (“I could not pay;” “I was not sure where to go to get medical care;” “I did not have transportation;” “Clinic’s hours of operation were not convenient;” “I was treated poorly at a clinic in the past;” “I did not want to be seen at a medical clinic;” “I don’t trust doctors;” “I didn’t have child care;” “I was too drunk or high;” “I was afraid they’d treat me with disrespect since I use drugs”).

^3^ Among participants reporting ever injecting drugs.

Supplemental Figures. Forest Plots of Regression Models with I^2^ scores of greater than 50%.


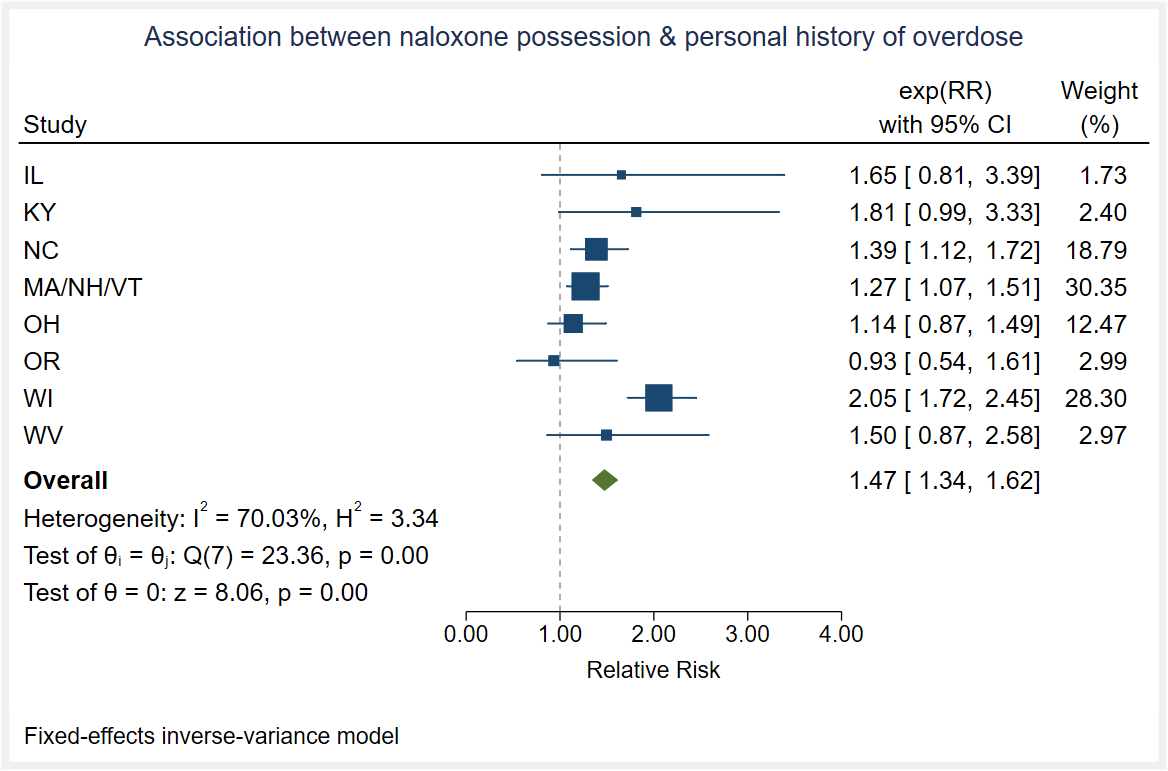


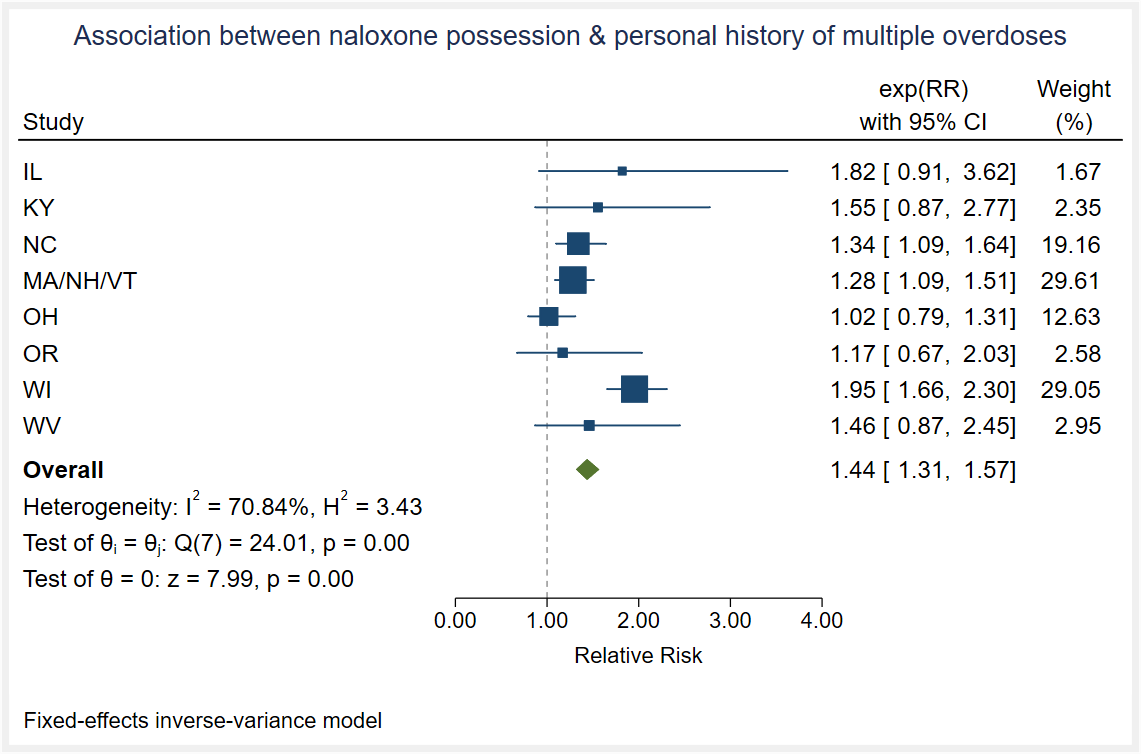


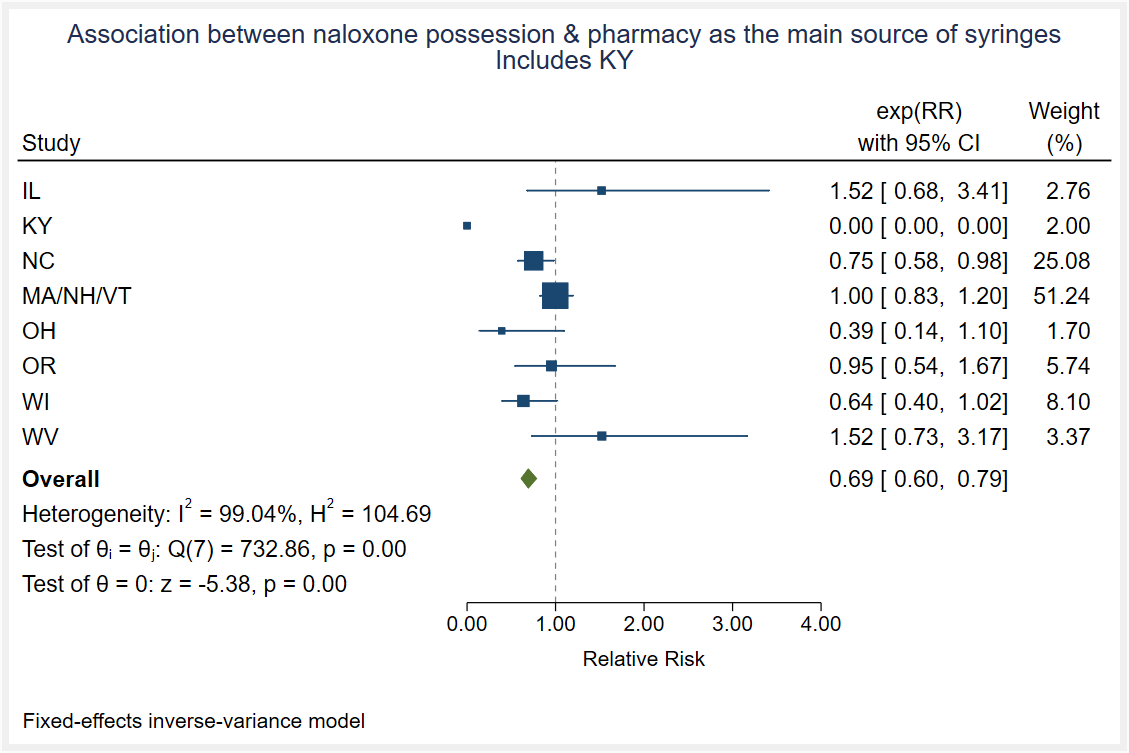


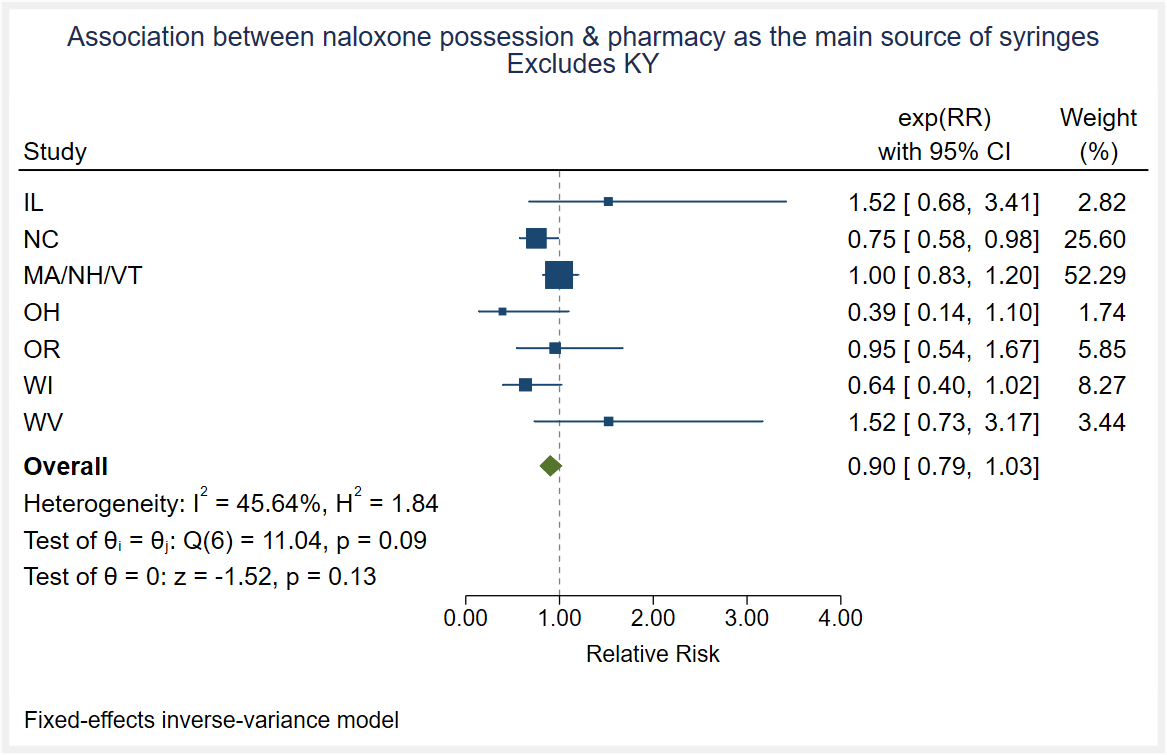


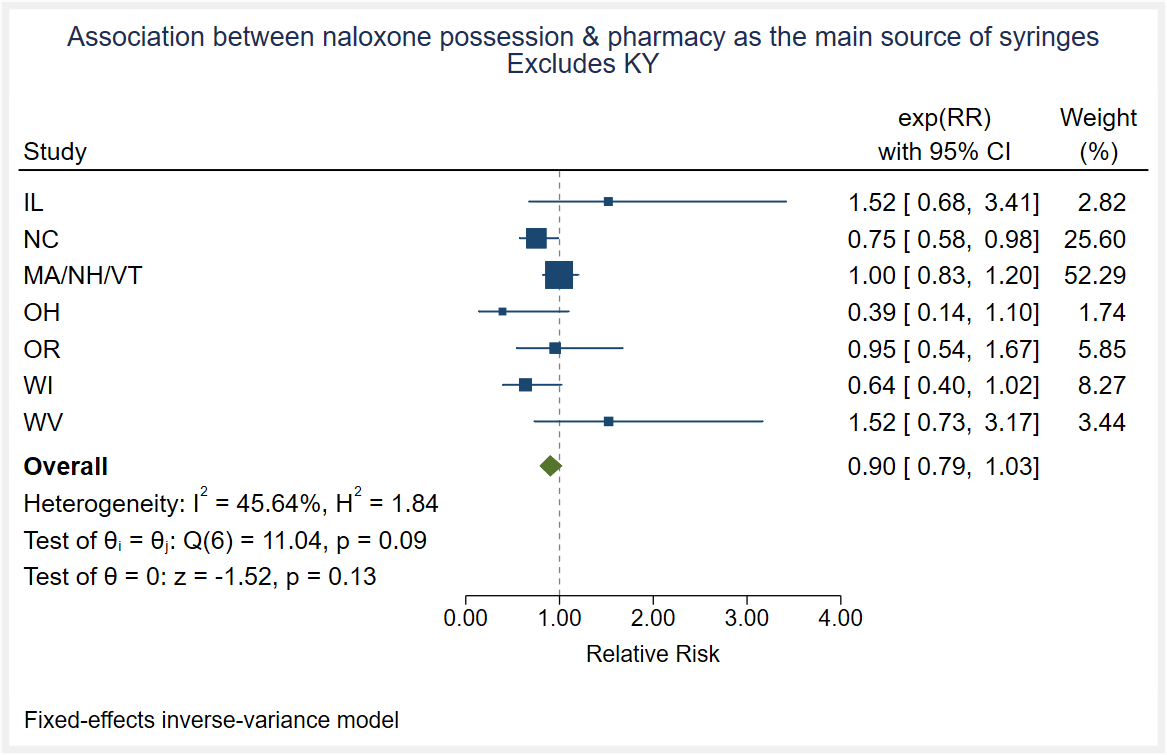

Supplement: Supplementary file 1 — Table S1: Multivariate associations of Rural Opioid Initiative participants who used any opioids in the past 30 days, by experience with overdose, access to care, substance use and injection drug use behaviors, addiction treatment and current naloxone possession status, adjusting for age, sex, and race/ethnicity (n = 2579) Supplemental Figures. Forest plots of regression models with I 2 scores of greater than 50%. [file JRH-42-0-s001.docx]
